# From chaos to control – experiences of healthcare workers during the early phase of the COVID-19 pandemic: a focus group study

**DOI:** 10.1186/s12913-021-07248-9

**Published:** 2021-11-10

**Authors:** Fredrik Rücker, Maria Hårdstedt, Sekai Chenai Mathabire Rücker, Emma Aspelin, Alexander Smirnoff, Anders Lindblom, Catharina Gustavsson

**Affiliations:** 1 Department of Infectious Diseases, Falun hospital, Infektionskliniken, Falu lasarett, SE-79182 Falun, Sweden; 2 Center for Clinical Research Dalarna - Uppsala University, Nissers väg 3, SE-79182 Falun, Sweden; 3 School of Medical Sciences, Faculty of Medicine and Health, Örebro University, SE-70182 Örebro, Sweden; 4 Vansbro Primary Health Care Center, Moravägen 27, SE-78633 Vansbro, Sweden; 5 Department of Infectious Control, Falu Hospital, Region Dalarna, Smittskyddsenheten, Falu lasarett, SE-79182 Falun, Sweden; 6 Unit of Infectious Diseases, Department of Medical Sciences, Uppsala University, Akademiska sjukhuset, Infektion, SE-75185 Uppsala, Sweden; 7 Department of Public Health and Caring Sciences, Uppsala university, BMC Box 564, SE-751 22 Uppsala, Sweden; 8 School of Health and Welfare, Dalarna university, SE-79188 Falun, Sweden

**Keywords:** Coronavirus disease 2019, Health personnel, Infection control, Mental health, Occupational health, Patient care, Qualitative research

## Abstract

**Background:**

The Coronavirus disease 2019 (COVID-19) pandemic has caused overwhelming challenges to healthcare systems worldwide. Healthcare workers (HCWs) have faced particular challenges: being exposed to the coronavirus SARS-CoV-2 and caring for patients having a new and potentially life-threatening disease. The aim of this study was to explore how HCWs in the Swedish healthcare system perceived their work situation during the first phase of the COVID-19 pandemic in 2020.

**Methods:**

Focus group discussions and interviews with HCWs were performed from June to October 2020 in one Swedish healthcare region. A purposeful sampling approach was used to select a variety of professions (physicians, nurses, nurse aides and cleaners) and workplaces (hospital inpatient wards, emergency department, nursing home and home care service). Qualitative content analysis was used for data analysis.

**Results:**

In total, 51 HCWs participated in eight focus group discussions and one HCW participated in an individual interview. The content analysis identified two main categories: ‘*Concerns about the risk of infection and transmission of infection to others’*, and ‘*Transition from chaos to managing in a new and challenging work situation’*. The findings revealed how HCWs perceived working conditions, including experiences of fear for personal health, confusion and uncertainty regarding personal protective equipment and infection prevention and control (PPE/IPC), and fear of infecting others. Both fearful and appreciating attitudes were achieved from the surrounding community. Helpful strategies for transition from chaos to control were lifted i.e. present and supportive leadership, and finding comfort and strength in the working team. Both helplessness and meaningfulness were described when caring for COVID-19 patients.

**Conclusions:**

This study provides unique insights into HCWs experiences of an extremely challenging work situation during the first phase of the COVID-19 pandemic, including feelings of stress and insecurity in a chaotic and hazardous working environment. But there is also mitigation of these challenges and even positive experiences including feelings of safety and meaningfulness. To enhance safety among HCWs in healthcare crises such as the COVID-19 pandemic, the findings highlight the importance of avoiding confusion about PPE/IPC, having a supportive healthcare leadership and ensuring accurate information provision about virus transmission to the public.

**Supplementary Information:**

The online version contains supplementary material available at 10.1186/s12913-021-07248-9.

## Background

The Coronavirus disease 2019 (COVID-19) caused by a previously unknown coronavirus, severe acute respiratory syndrome coronavirus 2 (SARS-CoV-2), was declared a global pandemic by the World Health Organisation (WHO) on March 11, 2020 [1]. The rapid virus transmission in Europe and USA in the spring of 2020 caused overwhelming challenges at all levels of the healthcare systems. Hospitals became overwhelmed with patients infected by the new virus and in Sweden the virus hit the elderly population hard [2]. The knowledge on treatment of patients and protection of healthcare workers (HCWs) from being infected was scarce. Personal protective equipment (PPE) for HCWs immediately became a global scarce commodity [3].

In Sweden, the Public Health Agency issued recommendations on PPE and infection prevention and control (IPC), but each of the 21 healthcare regions decided independently which PPE to be used by HCWs in that region [4]. The lack of evidence regarding the most effective PPE contributed to regional differences in use of PPE in the beginning of the pandemic. Initial shortage of available material also affected local differences in PPE use.

Early on during the pandemic, in the spring of 2020, several countries reported on high percentages of HCWs being infected by SARS-CoV-2 [5, 6]. Contributing factors were presumably lack of knowledge regarding virus transmission and unpreparedness in the healthcare organisation with inadequate PPE as well as lack of IPC training [7, 8]. Reports have concluded that HCWs caring for COVID-19 patients have increased risk of stress and burnout as well as mental health problems, including depression, anxiety, insomnia [9] and post-traumatic stress disorder [10, 11]. However, there is insufficient knowledge about how HCWs perceive their work situation during the pandemic. Studies based on survey data have attributed mental health problems in HCWs dealing with COVID-19 to fear of getting infected, fear of being a carrier and spreading the disease to others [12], stigmatization [13] and work overload [14]. The very few interview studies undertaken with HCWs to in-depth explore their experiences of working during the early phase of the pandemic, have focused on nurses and HCWs in emergency care [8, 15, 16]. To the best of our knowledge, there are no studies taking into account the multi-professional perspective of care by including all relevant health professions involved in the care of COVID-19 patients. In addition, there is a gap in knowledge concerning the experiences of HCWs at different levels of care, i.e. also including community-based care at nursing homes and home care services, in addition to inpatient hospital care.

The aim of this study was to explore how HCWs perceived their work situation during the first phase of the COVID-19 pandemic in 2020. We investigated the experiences of HCWs in different parts of the healthcare organisation: hospital inpatient wards and emergency department, as well as nursing home and home care service.

## Methods

### Study design

This study had a descriptive design, using qualitative content analysis of focus group discussions and one individual interview with HCWs [17, 18].

### Participants and setting

A purposeful sampling approach was used to select HCWs with experience of working with COVID-19 care in one Swedish healthcare region. The healthcare region had a catchment population of 270,000 and managed public inpatient and outpatient care. From early March to mid-April 2020 there was a sharp increase in patients with COVID-19 admitted to the main county hospital in the region responsible for all inpatient care of COVID-19 at the time. Patients with moderate to severe respiratory failure were cared for at the infectious disease ward, which had high-flow oxygen treatment available. If need for invasive mechanic ventilation the patients were transferred to the intensive care unit (ICU). Additional wards designated for COVID-19 care were opened and the ICU expanded, which increased fourfold the capacity for care of COVID-19 at the hospital. During the peak in mid-April, 85-90 % of all beds available at the infectious disease ward and the additional COVID-wards were occupied with COVID-19 patients and many patients with COVID-19 were treated at the ICU. A few patients were relocated to other hospitals due to lack of ICU capacity. The number of COVID-19 patients cared for at the hospital remained high until mid- July when the first wave started to cease. The majority of patients admitted to the hospital were 60-80 years. During the spring 2020, there were several outbreaks at nursing homes for the elderly and many of those patients were cared for at the nursing homes, and not admitted to the hospital. Many fragile elderly having home care service were also affected by COVID-19.

Due to shortage of staff, especially nurses and nurse aides, HCWs often had to work additional shifts during the study period. At the hospital, HCWs were relocated from other wards to cover for the care of COVID-19 patients. However, HCWs belonging to a risk group could ask to be transferred to other duties. A telephone helpline was started at the hospital in April 2020, where HCWs could turn for support if needed. At the infectious disease ward, debriefing groups were organized for nurses and nurse aides.

The sampling aimed to represent a variety of professions (physicians, nurses, nurse aides and cleaners), years of professional experience and different workplaces within the healthcare system, i.e. hospital inpatient wards and emergency department, as well as nursing home and home care service. Also, the sampling aimed to represent two different public care holders: hospital and emergency care are managed by the healthcare region while nursing homes and home care service are community based. Oral and written information about the study was given to all participants prior to the focus group discussions and written consent was obtained from all participants. The study was approved by the Swedish Ethical Review Board (ID-no 2020-03120).

### Procedure

Focus group discussions were performed from June to October 2020. Individual interviews were offered if, by any reason, the group format was not possible for the participant. The focus group discussions were led by a moderator and a note taker was present. The authors took turns to act as moderator and note taker during the interviews. The moderator used a semi-structured topic guide containing open-ended questions elaborated to respond to the research questions:

- How do HCWs perceive their work situation during the COVID-19 pandemic?
- What are the thoughts and feelings of HCWs concerning risk and safety when caring for COVID-19 patients?
- How do HCWs perceive IPC measures at the workplace, and risk of getting infected and of spreading COVID-19 to others?

The focus group format was chosen to promote interaction and information exchange between participants [19]. The moderator used the semi-structured topic guide to start and guide the discussion, adding probing and follow-up questions. The moderator made sure that all participants could participate actively in the discussion. It was taken under consideration that the authors’ affiliation with the healthcare region could possibly hinder the participants’ willingness to speak freely during discussions. Thus, the focus group moderator emphasised that participation was voluntary and that confidentiality of the spoken information was to be kept. The focus group session lasted until the moderator sensed that further discussion did not produce any new understanding of the research questions.

 The focus group sessions were carried out at the participants’ workplace and only the participants, moderator and note taker were present. The individual interview was undertaken in the same way. Special precautions were taken to avoid the risk of virus transmission during the focus group discussions. At the time for the focus group discussions, the Swedish IPC recommendations allowed group meetings to take place. The official recommendations concerning how to undertake group meetings to avoid virus transmission were followed: participants and moderators sat in a large well-ventilated room, at least one meter apart from each other and followed hand hygiene routines. Symptomatic participants and moderators were not allowed to participate. Physical contact, e.g. handshaking, was not allowed. In addition, online focus group meeting was offered in case the workplace could not accommodate a sufficiently large room to ensure sufficient space between participants. The sessions lasted for 30-70 min. The discussions were audio-recorded and transcribed verbatim after the sessions.

### Data analysis

The text files from the focus group discussions and the interview were analysed using qualitative content analysis with an inductive approach [17]. The analysis was descriptive, meaning that we aimed to stay as close as possible to the data when coding and interpreting the findings. The inductive analytic approach was deemed suitable to explore the HCWs experiences unconditionally. It allowed us to explore and describe the participants’ experiences and perceptions of the phenomena without any pre-set theoretical framework or preconceptions guiding the data analysis.

To increase trustworthiness of the study the quality criteria as described by Lincoln and Guba were followed [20]. Credibility was enhanced by a systematic and thorough analysis process, in which all authors were involved. The broad competence in the research group strengthened the analytical process and helped in facilitating the focus group discussions. The authors have experience of clinical work in the field of IPC and infectious diseases (FR, SCMR, EA, AS, AL) as well as experience of performing clinical research in healthcare settings (MH, SCMR, AL, CG) and of performing qualitative content analysis (CG). The text files were read through several times to identify meaning units, i.e. parts of the text containing information relating to the research questions. The meaning units were given code labels. The codes were grouped and organized into higher-order subcategories and categories. To validate the interpretation, the text files and codes were re-read by the authors and the organization of subcategories and main categories was reviewed until consensus was achieved. Table 1 provides an example of the coding process. NVivo software (Nvivo 1.3, QSR International Pty Ltd, Chadstone, Victoria, Australia) was used for the analysis.

**Table 1:** Examples of the coding process by examples of codes and meaning units in one subcategory in the main category ‘Concerns about the risk of infection and transmission of infection to others’

Main category	Subcategory	Examples of codes	Examples of meaning units
Concerns about the risk of infection and transmission of infection to others	Fear of contracting SARS-CoV-2	One nurse aid feels worried about his/her personal health when previously healthy persons are admitted to the ward	‘*At first I thought that these risk groups, well, they have these underlying conditions. But when someone is admitted who is healthy, and not old, and do not belong to a risk group and becomes seriously ill, then you come to relate to it more personally – it could have been me, it could have been my best friend!*’
		Initially fear of being infected by COVID-19 while cleaning on the COVID-19 ward but the fear decreased thanks to clear infection control instructions by the ward staff	’*I was afraid in the beginning, of cleaning patient rooms in the COVID-19 ward. I was afraid of getting infected but after a while I got used to it. I got good directives from the ward staff regarding infection control procedures*.’

COVID-19: Coronavirus Disease of 2019

## Results

In total, 51 HCWs participated in eight focus group discussions (Table 2). In addition, one interview was undertaken with a nurse who, due to practical reasons, could not attend the scheduled focus group discussion. At the infectious disease ward, four focus group discussions divided by professions: physicians, nurses, nurse aides and cleaners, were performed. One focus group was performed with nurses and nurse aides at an inpatient ward designated for COVID-19 care. One focus group was undertaken with nurses and nurse aides at an emergency department. One focus group was undertaken with nurses, nurse aides and a first line manager at a nursing home. One focus group was undertaken with nurse aides working in home care service. All focus groups were composed of participants having varying professional experience before the COVID-19 pandemic, from newly graduated to more than 40 years of experience.

**Table 2:** Participants in the focus group discussions (*n*=51) and the individual interview (*n*=1)

Focus group number	Workplace	Number of participants	Professions	Date for discussion month/day/year
1	Inpatient infectious disease ward	3	Physicians	6/18/2020
2	Inpatient infectious disease ward	6	Nurses	6/17/2020
3	Inpatient infectious disease ward	7	Nurse aides	6/11/2020
4	Inpatient infectious disease ward	7	Cleaners	6/26/2020
5	Inpatient ward designated COVID-care	8	3 Nurses; 5 Nurse aides	7/23/2020
6	Emergency department	7	2 Nurses; 5 Nurse aides	7/29/2020
7	Nursing home	8	2 Nurses; 5 nurse aides; one first line manager	9/23/2020
8	Home care service	4	4 Nurse aides	10/12/2020
Individual interview				
1	Inpatient infectious disease ward	1	Nurse working night shift	6/17/2020

The qualitative content analysis identified two main categories: ‘*Concerns about the risk of infection and transmission of infection to others’* and ‘*Transition from chaos to managing in a new and challenging work situation’*. The categories and subcategories are illustrated in Tables 3 and described in further detail below, with quotations from the focus groups in italics.

**Table 3:** Results of the qualitative content analysis of focus group discussions and one individual interview with healthcare workers (*n*=52)

Main categories	Subcategories	Code groups
Concerns about the risk of infection and transmission of infection to others	Fear of contracting SARS-CoV-2	Experience of seeing patients severely ill with COVID-19 increased the fear of being infected
		Concerns about getting infected at work
		Thinking rationally about the risk of infection and risk of severe disease as a way to deal with the fear of infection
	Confusion and uncertainty about IPC routines and proper PPE	Fear that the IPC measures and PPE are inadequate to protect from infection
		Fear that inappropriate use of the PPE may increase the risk of infection
		Fear of shortage of PPE
		The PPE is an obstacle during patient care
		Repeated changes in IPC routines, including PPE
		Differences in IPC routines, such as PPE and staff testing, between workplaces
		Positive experiences of IPC routines and PPE
	Fear of transmitting SARS-CoV-2 to others	Fear of transmitting SARS-CoV-2 to close family and relatives
		Fear of transmitting the virus to patients, colleagues and other work-places
	Both fearful and appreciating attitudes from the surrounding community towards HCWs	The HCWs being stigmatised due to that they worked with COVID-19
		Family members fear that the HCW will get infected with COVID-19
		Positive response from the surrounding community
	Frustration about the media coverage of the COVID-19 pandemic	Media contributed to fear of transmission of virus and stigmatisation of HCWs
		HCWs have felt forced to deal with or address the exaggerated image of the pandemic that the media created
Transition from chaos to managing in a new and challenging work situation	The healthcare leadership transition: stepping up or stepping back in response to the crisis	Initial chaos in the healthcare organisation
		A completely new situation demanded radical and immediate changes in management and work routines at all levels in an unprepared organisation
		Inadequate top-down information provision from the central healthcare organisation to the local management and further down to the HCWs
		The management’s ability to lead during a crisis: being present versus abandoning the HCWs
		The extreme work load contributed to a more patient-centred work situation and less administrative duties
	Mixed feelings of helplessness and frustration versus meaningfulness and pride when caring for COVID-19 patients	Feelings of helplessness when being close to very sick and suffering patients
		Challenging and stressful to work with new medical technology and more advanced level of care without proper training
		Troublesome communication with the patients’ relatives
		Feeling satisfaction and pride in doing an important and meaningful work
		Finding comfort and strengths in the working team

COVID-19: Coronavirus Disease of 2019, SARS-CoV-2: Severe Acute Respiratory Syndrome Coronavirus 2, IPC: Infection Prevention and Control, PPE: Personal Protective Equipment, HCW: Healthcare Worker

### Concerns about the risk of infection and transmission of infection to others

#### Fear of contracting SARS-CoV-2

In all focus group discussions, the HCWs expressed fear of contracting SARS-CoV-2. Specifically, they worried about being infected at work since they knew that the virus would be present at the workplace, certainly among patients and perhaps among co-workers. Several participants expressed that caring for patients with critical COVID-19 infections increased their fear of infection and of becoming seriously ill.

‘*At first I thought that these risk groups, well, they have these underlying conditions. But when someone is admitted who is healthy, and not old, and do not belong to a risk group and becomes seriously ill, then you come to relate to it more personally – it could have been me, it could have been my best friend!*’ (Nurse aide, emergency department).

There was in general less fear of infection among the HCWs that had previously worked with infectious diseases. Those participants gave possible explanations to why they may be less worried of being infected: IPC training leading to an understanding of and adherence to IPC, the infectious disease ward being designed to control the spread of infectious diseases and experience in treating patients with other potentially hazardous contagions. Some participants described that thinking rationally about the risk of infection and severe disease was a way to deal with the fear of infection.

Participants also expressed that feelings of safety at work could actually put them at risk. One common experience was that although HCWs were vigilant when caring for COVID-19 patients, they often had a false sense of safety outside of the patient room, forgetting about safety precautions, such as keeping distance to colleagues.

#### Confusion and uncertainty about IPC routines and proper PPE

Fear that the IPC measures were inadequate to protect from infection was a concern in all focus group discussions. The participants described that in the beginning of the pandemic everyone, including experts, were uncertain about what were adequate IPC and PPE. This uncertainty was described as very frustrating. Several of the participants expressed fear that the PPE would not be adequate to protect them from infection. Factors mentioned that contributed to this fear were: different PPE used at different workplaces, too short face shields, substandard facemasks that were later recalled, and short-sleeved aprons that did not protect the arms.

*‘It felt very uncertain in the beginning, no one really knew how to dress [in the PPE]. In other units they used other sets of PPE. What is enough if someone is coughing? I was very uncertain if they knew what was needed to protect us. Aerosols, for example… a lot was unknown. We didn’t know what we were getting ourselves into. It felt very unsafe, the uncertainty that no one really knew. The uncertainty, that the physicians didn’t know, we didn’t know, not even the management at the top of the organisation knew.’* (Nurse aide, COVID-19 ward).

Differences in IPC routines between workplaces, led to fear that lack of evidence or shortages of PPE caused these differences. Also, the participants’ reasoned that if IPC were different in two workplaces, one of these workplaces had to be less safe to work at. The differences in PPE became frustratingly evident when HCWs from different workplaces met to hand over a COVID-19 patient, e.g. home care service staff handing over to the ambulance staff.

*‘…aprons and thin face shields was all we had and they [the ambulance crew] gave us strange looks. They [the ambulance crew] looked like astronauts, they were covered from tip to toe.’* (Nurse aide, home care).

Recommendations of IPC routines including PPE were constantly changing over time, sometimes from day-to-day, which was described as tiring and stressful by some participants.

*‘There was so much contradicting information for a while. We had new infection control routines every other hour. Of course that caused irritation.’* (Nurse, infectious disease ward).

Participants described conflicts between staff, as some HCW started using more PPE than was recommended while others followed the official recommendations.

*‘At our ward there was no consensus, there were many strong opposing opinions. Some said that it should be long sleeves and some wanted all the equipment although we were not supposed to use that according to the official directives.’* (Nurse aide, nursing home).

The participants in one focus group had experienced an outbreak of COVID-19 at the same time as facemasks started being used, and subsequently were unsure if it may have been an inappropriate use of facemasks that facilitated the spread of the virus.

Lack of PPE was a major problem in the beginning of the outbreak especially for participants working in the nursing home and in home care service. At one of these workplaces, the HCWs had produced their own PPE due to lack of supply. Participants from hospital wards and emergency department did not experience lack of PPE, but rumours that PPE routines omitted essential equipment to save material created mistrust in official PPE recommendations. When other HCWs were given personal military grade facemasks as backup in case single use facemasks would run out, the cleaning staff working at the same ward were not offered such masks.

Some participants expressed that the PPE and other IPC made the working environment worse and was an obstacle during patient care.

*‘And to work with these face shields and sometimes respirators… I can’t see well, it gets foggy. I have been stressed, in a different way than I usually do. It gets sweaty and foggy and I can’t insert an intravenous cannula because I can’t see anything, and the face shield is in the way.’* (Nurse, infectious disease ward).

Besides from PPE use, other concerns regarding the risk of transmission at work were mentioned: lack of social distancing in the workplace, insufficient adherence to IPC routines because of ignorance or stress, and wards having inappropriate spaces to provide satisfying conditions for infection control.

*‘In the lunchroom we don’t sit that very close, but the nurses’ stations, they are crowded… and in the nurses’ medicine storage room. Some rooms are just difficult to… distance yourself in. We can’t move away from each other since the computers are set where they are, so there is no choice but to sit in those cramped nurses’ stations ’* (Nurse, COVID-19 ward).

Participants working at the nursing home expressed that the particular difficulty for residents with dementia to adhere to IPC routines and social distancing caused dangerous situations for both residents and HCWs.

Regarding diagnostic testing of SARS-CoV-2, the cleaners had experiences of not getting access to testing at the hospital when they had symptoms, although all other HCWs working at the same ward were offered testing at work. One cleaner was told to book an appointment for PCR-testing at the primary healthcare centre, which meant a delay in testing for several days. She also had to pay for the test although it was supposed to be free according to Swedish law.

*‘…it sucks that you have to pay for something that you were exposed to at work.’* (Cleaner, infectious disease ward).

There were also some participants that were comfortable with the IPC routines and PPE. They expressed that having knowledge of transmission routes and IPC principles is more important than the actual PPE. Many participants felt more comfortable with the IPC and PPE over time when knowledge of transmission routes increased and they did not become infected. Participants that were used to work with infectious diseases before the COVID-19 pandemic were more likely to have trust in IPC routines and PPE.

*‘I would not have been able to work at the infectious disease ward for 15 years if I had been afraid of getting or transmitting infections, whether it is COVID-19 or another disease. If you are afraid of infections I believe you look for work elsewhere.’* (Nurse, infectious disease ward).

#### Fear of transmitting SARS-CoV-2 to others

In all focus groups, participants expressed fear of transmitting SARS-CoV-2 to their close family and relatives, and many had taken precautions exceeding official recommendations to ensure that they did not infect their relatives. Some participants were worried that they were carriers of the virus at all times and isolated themselves from family members such as partners and children. Participants described how they were aware of the risk of transmitting the virus to others: patients, colleagues and staff at other workplaces and hospital wards. One participant described this risk as more stressful than the risk of getting infected herself.

#### Both fearful and appreciating attitudes from the surrounding community towards HCWs

In all focus groups, the HCWs expressed that they felt stigmatised due to their work with COVID-19 patients. Stigma was most commonly described as fear and avoidance by friends and neighbours. Some participants described how their children were no longer allowed to play with their friends because the parents were afraid of COVID-19.

*‘…as if we have the plague. For example, my children, […] they haven’t been welcomed to their friends’ houses because of where I work. People think there is a risk of transmission of the infection.* (Nurse, infectious disease ward)

Several participants described that their wellbeing was affected by being socially isolated. This was a result of stigma and self-imposed isolation, in addition to the social distancing rules and recommendations in the general society. Some participants had family and friends that did not understand the working conditions of the HCWs, which contributed to a sense of loneliness. No participants described that they felt stigmatised by their closest family, but many expressed that family and relatives were worried that they would get infected.

 However, some of the participants had experienced positive responses from people in the surrounding community. One common example was free food and snacks being sent from restaurants and companies as a sign of support and appreciation. Some participants said that their relatives and friends expressed admiration, pride and curiosity of the hard work that HCWs were doing during the pandemic.

#### Frustration about the media coverage of the COVID-19 pandemic

The media coverage of the COVID-19 pandemic was perceived by some participants as exaggerating the risk of virus transmission to HCWs in healthcare settings. Thus, increasing fear and stigmatization of HCWs as carriers and spreaders of COVID-19. Some participants have felt forced to deal with or address what they perceived as criticism towards HCWs in media.

*‘Something that I have felt stressful is the media. Outside of work, I have felt questioned. Where I live, media has reported about inadequate protective equipment at nursing homes and things like that. …and when there has been stuff on social media about patient cases, I have felt criticised.’* (Nurse, infectious disease ward).

### Transition from chaos to managing in a new and challenging work situation

#### The healthcare leadership transition: stepping up or stepping back in response to the crisis

Some participants described a chaotic start of the pandemic at their workplace. This was attributed to several factors, but most notably the unpreparedness of the healthcare organisation for such a large-scale crisis. Some participants described how they, together with the whole healthcare organisation, initially underestimated the COVID-19 pandemic. This was something that quickly changed on a personal level when the first critically ill patients were admitted to the hospital. Some participants expressed a sense of panic, a feeling that the healthcare system would not be able to withstand the pressure of the pandemic.

*‘Every patient was so sick. Then, one of the staff became critically ill and was taken to the intensive care unit. Then I felt like ’Oh no, we will never manage. This can affect anybody, it is just too frightening’* (Nurse, infectious disease ward).

In all focus groups, participants expressed that it was often difficult to keep up with the extensive flow of information on changes in routines coming from the management. However, most participants thought that this improved over time when changes in routines were not as frequent and when managers organised systems to disperse information, such as easily accessible protocols and regular staff meetings. Regarding PPE, nursing home and home care staff were less content with the management’s provision of information compared to hospital staff. Cleaning staff at the hospital, who cleaned at the COVID-19 wards but belonged to a separate organisation, expressed that they were sometimes not informed about updated IPC routines. Lack of information led to that cleaning staff were accidently exposed to SARS-CoV-2 at the wards, e.g. they were not informed about the use of high-flow nasal oxygen therapy in patient rooms with risk for aerosols. Actually, the cleaning staff expressed that the greatest challenge for them during the pandemic had been insufficient information about IPC from their management. They also described that information coming from different parts of the organisation was often conflicting and thus confusing.

*‘It was very unclear to us. The notice-board for cleaning information in the infectious disease ward says that we should use facemasks, so we do that. Then our manager comes and says no, you shouldn’t wear those. Then someone else comes and says that we should wear them. It has been a lot like that.’* (Cleaner, infectious disease ward).

In all focus groups, the participants underscored the importance of supportive leadership in a crisis and a demanding work situation. In particular, this concerned the first line management’s ability to be present for supporting the HCWs. Some of the participants expressed that their first line managers were important for finding comfort in the new and challenging work situation, pointing to the managers’ ability to restore order in chaos, provide information, and addressing concerns and worries within the group. Others had felt abandoned by their first line management. Those HCWs felt that they had to deal with the demanding work situation all by themselves, while the management escaped the risk of infection by staying in secure offices.

*‘The managers were sitting in protected places, in offices. To protect themselves. Yeah, they should protect themselves but we shouldn’t! We were supposed to go out there and work.’* (Nurse aide, home care).

Both of these perspectives underscored the importance of physical presence of first line managers at the workplace. The most supportive managers had even occasionally helped with the patient care, thereby showing solidarity and understanding of what the HCWs were going through in terms of risk of infection and heavy workload. Furthermore, not being physically present made it more difficult for the manager to assess the workload for the staff.

*‘I was in contact with my boss, and after a while I just wanted to throw away the phone. The boss said – ’Hey, didn’t you have time to fix this?’ I replied ’Did you know that we are four staff short today? Do you think I have time to sit down at all?’ I had to stay until 9 pm and beg other staff to stay. Then the boss said ’You have to go and fix this’. I responded ’Listen, I just can’t.’* (Nurse aide, home care).

Several participants, especially at the hospital, had positive experiences of the organisational transition made by the healthcare system at the beginning of the COVID-19 pandemic. Some were pleasantly surprised and impressed by the quick transition at an organisational level. Several participants described the work during the pandemic as more patient-centred and comprising less administrative duties than before the pandemic. In most focus groups, the participants mentioned that the pandemic contributed to an improved, less competitive and less prestigious communication between different hospital departments units, but also between professions such as nurses and physicians.

*‘…the teamwork has been something very positive. This includes colleagues at other clinics, it has been very special in that way.’* (Physician, infectious disease ward).

#### Mixed feelings of helplessness and frustration versus meaningfulness and pride when caring for COVID-19 patients

Many participants expressed feelings of helplessness and guilt when caring for COVID-19 patients. This was especially evident for nurse aides and nurses in the hospital, nursing home and home care service who spend time close to the patients. These feelings were less evident among physicians, and not at all expressed by cleaners.

*‘We feel that it is terrible, since we have a close relationship to our clients and know them so well.’* (Nurse aide, home care).

The general lack of knowledge concerning treatment and disease progression early in the pandemic created moral stress among HCWs. The participants described helplessness of not being able to provide adequate help and comfort for the patients. Physicians expressed frustration over not having better treatments for the patients. Furthermore, working with new medical technology such as high-flow nasal oxygen equipment and a more advanced level of care without proper training, was challenging and stressful for several of the participants. Many participants commented on the fact that COVID-19 patients are quite lucid despite being critically ill, which is less common in other critical conditions. The patients asked questions about their condition and understood how serious the situation was. This fact was perceived to increase the suffering of the patients, thereby adding to the moral stress of the HCW working close to the patients.

*‘The COVID-19 patients are so very conscious. Our normal sepsis patients, their mental state is affected by hypotension so when they have to go the ICU they are barely aware of what is happening. The COVID-19 patients on the other hand are completely awake. They talk to their relatives on the phone, informing them that they are about to be transported to the ICU for ventilator care. That is the hardest thing for me.’* (Nurse, infectious disease ward).

In most focus groups, the participants described an increased workload during the pandemic and some described symptoms of stress and burnout, such as chronic tiredness, irritability and stomach pain. This was especially true for nurse aides and nurses in the hospital and in the nursing home and home care.

*‘I couldn’t sleep some evenings because of everything I had been through during the day. I had dreams the whole night. Then I had to go back to work, to the same chaos.’* (Nurse aide, home care).

Patients’ relatives were generally not allowed at the wards, which made communication with relatives an important and time-consuming part of COVID-19 care for HCWs. This extra workload was stressful. Frustrated, suspicious relatives were perceived troublesome to handle for some of the participants.

Some participants reflected on the positive side of treating COVID-19 patients: a sense of meaningfulness and pride of their work. This feeling was enhanced by the fact that the COVID-19 pandemic allowed the HCW to focus less on administrative duties and more on patient centred care.

*‘One positive thing was that we were together working as a team. The doctor joined us and did not have to leave to see other patients in the outpatient department. There were no distractions for the rest of us as well, no administrative work. Only patient care, and that brought us together. Nurse aide, nurse and doctor next to these patients the whole shift.’* (Nurse, infectious disease ward).

Many participants pointed out that the team spirit had been stronger than ever during the pandemic and that HCWs supported each other more. A common will to help out with COVID-19 patient care was shared by several of the participants. Some expressed frustration that they had to stay at home when they had COVID-19, because they knew how much work needed to be done at the workplace and they really wanted to help out.

*‘You feel appreciated from all levels, managers, physicians, anaesthesiologists, everybody asks how you are feeling. The hospital church as well. Everybody makes an effort to show each other appreciation.’* (Nurse aide, COVID-19 ward).

## Discussion

This study presents a unique and rich material based on focus group discussions with HCWs performed close in time to the first phase of the COVID-19 pandemic in Sweden in the spring of 2020. The results include experiences from a broad range of HCWs both working in hospital inpatient wards and the emergency department, as well as a nursing home and home care service. The findings provide in-depth exploration of the HCWs experiences and perceptions of working with COVID-19 patient care in a challenging work situation during the pandemic outbreak. The participants express perceptions of fear for personal health, confusion and uncertainty regarding PPE and IPC routines, and fear of transmitting infection to others. They describe both fearful and appreciating attitudes from the surrounding community. They give examples of successful and unsuccessful leadership strategies in crisis management. Both feelings of helplessness and meaningfulness were described when caring for COVID-19 patients. A deeper understanding of how HCWs were affected during this pandemic can help in reducing experiences of stress and insecurity, and increasing experiences of safety among HCWs, even in a healthcare crisis where essential knowledge and resources are lacking. We believe the findings in this study are of relevance also in future similar scenarios.

Fear of SARS-CoV-2 infection was common among the participants and exacerbated by confusion and uncertainty regarding proper PPE and IPC. Also others have reported on fear of SARS-CoV-2 infection among HCWs. One study reported that a majority of HCWs were afraid of SARS-CoV-2 infection and 89 % believed that they were more susceptible to get COVID-19 compared to non-HCWs [21]. Participants with previous experience of working with infectious diseases had higher confidence in PPE and IPC, suggesting it beneficial with experience of working with hazardous infections and IPC routines prior to the COVID-19 pandemic. These participants expressed that it was more important to have knowledge of transmission routes and IPC principles then the actual PPE. We suggest that having HCWs with previous experience of working with infectious diseases supporting unexperienced HCWs is important to increase the confidence in IPC routines and reduce fear of infection. This is supported by an interview study where less experienced nurses emphasised the importance of emergency training and knowledge of infectious diseases for coping with their work during the pandemic [15].

Differences in IPC and PPE routines between different workplaces and care holders were of great concern for the participants. This contributed to speculations of inequality between workplaces and about the safety of different IPC routines. We suggest that IPC routines should be harmonised to avoid speculation about inequality among HCWs and about the safety of different IPC routines. There were also differences in availability of information and PPE supply between different workplaces and categories of HCWs. For example, the cleaners had difficulties receiving information about changes in IPC routines at the ward. They were not offered backup multiple use facemasks in case the single use masks would run out and some cleaners described limited access to SARS-CoV-2 testing. Notably, a recent Cochrane evidence synthesis concluded that it is important to include healthcare support staff such as cleaners when implementing IPC guidelines [22]. HCWs in the community based home care service and nursing home experienced a shortage of PPE at the beginning of the pandemic and had to work without PPE or use homemade PPE, while HCWs in the hospital described that they never had shortage of supply. We believe that several of these inequalities were related to lack of communication between the management in different organisations as well as lack of one common chain of planning and delivery of PPE supply for all care holders. For example cleaners, worked in the same ward as nurses and physicians but belonged to a different organisation of management at the hospital. Being aware of and harmonising information and supply of material between different organisations and care holders would reduce inequalities and speculations.

Participants described stigma facing themselves and family members because of their work with COVID-19 patients. This result corresponds to the findings in a survey in the United States and Canada during the early phase of the COVID-19 pandemic, showing that fear and avoidance of HCWs was expressed by more than one third of responders in the general population [13]. The HCWs in our study expressed fear of transmitting SARS-CoV-2 to patients, relatives and friends. They also conveyed difficulties in sharing traumatic experiences at work with their relatives. Altogether, the HCWs described a situation of physical and mental isolation in combination with a challenging work situation. The participants perceived that both the traditional and social media, exaggerated the risk of virus transmission and increased fear in the community, which contributed to frustration and stress among the participants. Emerging evidence suggests that almost a third of non-HCWs in the general population believe that HCWs are likely to have COVID-19 [13]. It is crucial for media to be aware of how public information can contribute to stigmatization and the negative impact it has on HCWs and the wider community.

The healthcare organisation’s unpreparedness for a large-scale pandemic was lifted in all focus group discussions. As a consequence of the COVID-19 pandemic, the management’s ability of leadership in a crisis became apparent to the HCWs. Participants in some focus groups described how the first line managers worked side by side with the HCWs, which was considered as very positive. In other focus groups, participants described how first line managers were physically absent from the workplace. The workload was described as extremely high and chaotic at times, and the HCWs perceived that the distant managers did not fully understand the situation. A recent literature synthesis emphasises that healthcare managers who lead with empathy provide comfort and stress reduction among HCWs during the chaotic working conditions of the COVID-19 pandemic [23]. We believe that managers that are physically present at the workplace signals solidarity and risk sharing in a stressful and hazardous working environment and increases the trust in IPC and in the management.

Moral stress was more evident among participants having professions working close to the patients, such as nurses and nurse aides, and less evident among physicians and cleaners. Foremost, this moral stress concerned the lack of knowledge of treatment and disease progression in the early phase of the pandemic, leading to feeling helplessness of not being able to provide adequate help for the patients. By contrast, the participants also expressed feelings of meaningfulness and pride in their work. Such positive feelings could plausibly be encouraged by managers as a means to mitigate moral stress among HCWs. In several focus groups, the participants described how the pandemic had brought an increased team spirit to the workplaces. The extreme workload resulted in a more patient-centred work situation with less administration duties, which strengthened the team. An Italian study of nurses’ experiences during the COVID-19 pandemic suggest that promoting team spirit is important to prevent conflict and stress in the workplace [16].

### Strengths and limitations

It is a major strength of this study that all focus discussions and the interview were performed during, or time wise close to, the first phase of the COVID-19 pandemic in Sweden in 2020.

To increase trustworthiness of the study the quality criteria as described by Lincoln and Guba were followed [20]. The broad competence in the research group; i.e. experience of clinical work in the field of infectious diseases as well as experience of performing clinical research in healthcare settings, strengthened the analytical process.

Recruiting participants of different professions, years of professional experience and different workplaces obtained a rich data set for confirmability. This study included participants that represented all relevant healthcare professions. Some HCWs declined to participate and gave lack of time as a reason. It cannot be ruled out that eligible HCWs declined to participate in the study due to very negative experiences of working during COVID-19-pandemic, thereby affecting the result of the study.

The results from data collection by qualitative methods and purposeful sampling are not assumed to be generalizable. The experiences expressed in the results of this study are contextual and situational which limits the transferability. However, the authors believe that the results may to some extent be transferrable to other HCWs working during the COVID-19 pandemic and also to other health crises in similar settings.

## Conclusions

This study provides unique insights into HCWs experiences of an extremely challenging work situation during the first phase of the COVID-19 pandemic outbreak in Sweden, including feelings of stress and insecurity in a chaotic and hazardous working environment. But there is also mitigation of these challenges and even positive experiences including feelings of safety and meaningfulness. To reduce stress and enhance safety among HCWs in healthcare crises such as the COVID-19 pandemic, the findings in this study highlight the importance of avoiding confusion about PPE and IPC, having a present and supportive healthcare leadership and ensuring accurate information provision about virus transmission to the public.

## Supplementary information

Additional file 1. The semi-structured topic guide used to direct the focus group discussions and individual interview (translated to English).

## Data Availability

The datasets generated and analysed during the current study are not publicly available as they consist of quotes by the interview subjects that might contain information, which could reveal the identity of individuals. But datasets with coded data are available from the corresponding author upon reasonable request.

## Abbreviations

COVID-19: Coronavirus Disease of 2019
HCW: Healthcare Worker
IPC: Infection Prevention and Control
PPE: Personal Protective Equipment
SARS-CoV-2: Severe Acute Respiratory Syndrome Coronavirus 2
