# Supplementary material for: From chaos to control – experiences of healthcare workers during the early phase of the COVID-19 pandemic: a focus group study
Source: BMC Health Serv Res. 2021 Nov 10;21:1219. doi: 10.1186/s12913-021-07248-9 (PMC8579171; doi:10.1186/s12913-021-07248-9)
Supplement: Supplementary file 1 — The semi-structured topic guide used to direct the focus group discussions and individual interview (translated to English). [file 12913_2021_7248_MOESM1_ESM.docx]

**Additional file.** The semi-structured topic guide used to direct the focus group discussions and individual interview (translated to English)

**Note:** As a rule, the moderator only asked the first level questions. The second level questions were noted in the guide as an aid to get the participants to elaborate some more on the topic when the response to the first level question was very brief.

**Introductory question:**

- First, can you briefly tell us about your role at the workplace and the tasks you have on a typical day?

**During this session we want to discuss about your experiences of working in healthcare during the COVID-19 pandemic:**

**-** What are your thoughts about your work situation during the COVID-19 pandemic?

- - How do you feel about your work during the pandemic?
  - How have your work situation or aspects of your work situation changed as a consequence of the pandemic?
  - What are the challenges you have experienced in connection with this change?
  - How has the workload been affected as a consequence of the pandemic?

**-** What are your thoughts and feelings about risks and safety when caring for COVID-19 patients?

- - What do you think and feel about the risk of getting COVID-19 yourself?
  - What are your thoughts on the consequences of falling ill with COVID-19?
  - Have you felt anxious or afraid of falling ill yourself and becoming seriously ill?
  - How have these thoughts and feelings changed over time?

**-** What are your thoughts and feelings about the risk of spreading the infection to others, such as patients, colleagues and relatives?

- - Have relatives expressed concern that you are exposed to the coronavirus at your workplace? If so, how has that affected you?
  - What reactions have you encountered from relatives and the rest of society based on your work with COVID-19 patients? Have these reactions changed over time?

**-** What are your experiences of the measures for infection prevention and control at your workplace during the COVID-19 pandemic?

- - What are your thoughts about the equipment and routines for infection prevention and control that have been recommended at your workplace?
  - Do you feel that there has been a consensus in the workplace regarding routines for infection prevention and control?
  - What do you feel about the information provided at the workplace regarding infection prevention and control during the pandemic? Enough information? Too little, too much?
  - What are your thoughts about that the recommendations regarding the personal protective equipment have changed several times during the COVID-19 pandemic?
  - Have you felt safe in the workplace or do you feel that you have exposed yourself to health risks at your workplace?
  - Have you felt a wish to use more or less personal protective equipment?
  - Have you been worried that the personal protective equipment will run out?
  - Have you felt a wish to change job/workplace or to change work-tasks at your workplace?

**-** Do you feel that some things should have been done differently in your workplace during the COVID-19 pandemic? If so, what and how?

**-** Is there anything else that you want to comment or add regarding your work situation during the pandemic?
